# Next-Generation Sequencing for Rodent Barcoding: Species Identification from Fresh, Degraded and Environmental Samples

**DOI:** 10.1371/journal.pone.0048374

**Published:** 2012-11-07

**Authors:** Maxime Galan, Marie Pagès, Jean-François Cosson

**Affiliations:** 1 Institut national de la recherche agronomique, Joint Research Unit Centre de Biologie pour la Gestion des Populations (INRA/IRD/Cirad/Montpellier SupAgro), Campus International de Baillarguet, Montferrier-sur-Lez, France; 2 Laboratoire de génétique des microorganismes, Institut de Botanique, Université de Liège, Liège, Belgium; Fordham University, United States of America

## Abstract

Rodentia is the most diverse order among mammals, with more than 2,000 species currently described. Most of the time, species assignation is so difficult based on morphological data solely that identifying rodents at the specific level corresponds to a real challenge. In this study, we compared the applicability of 100 bp mini-barcodes from cytochrome *b* and cytochrome *c* oxidase 1 genes to enable rodent species identification. Based on GenBank sequence datasets of 115 rodent species, a 136 bp fragment of cytochrome *b* was selected as the most discriminatory mini-barcode, and rodent universal primers surrounding this fragment were designed. The efficacy of this new molecular tool was assessed on 946 samples including rodent tissues, feces, museum samples and feces/pellets from predators known to ingest rodents. Utilizing next-generation sequencing technologies able to sequence mixes of DNA, 1,140 amplicons were tagged, multiplexed and sequenced together in one single 454 GS-FLX run. Our method was initially validated on a reference sample set including 265 clearly identified rodent tissues, corresponding to 103 different species. Following validation, 85.6% of 555 rodent samples from Europe, Asia and Africa whose species identity was unknown were able to be identified using the BLASTN program and GenBank reference sequences. In addition, our method proved effective even on degraded rodent DNA samples: 91.8% and 75.9% of samples from feces and museum specimens respectively were correctly identified. Finally, we succeeded in determining the diet of 66.7% of the investigated carnivores from their feces and 81.8% of owls from their pellets. Non-rodent species were also identified, suggesting that our method is sensitive enough to investigate complete predator diets. This study demonstrates how this molecular identification method combined with high-throughput sequencing can open new realms of possibilities in achieving fast, accurate and inexpensive species identification.

## Introduction

Because species are the basic unit of many fields in biology [1], [2], accurate species identification is an absolute prerequisite for studies focusing on agronomy [3], [4] human health [5], conservation biology [2], [6], ecology and evolution [7]. Whether pests, disease reservoirs or endangered species are considered, fast and accurate species identification is required for an ever increasing number of animal and plant samples [8].

Classical approaches to identification have traditionally been based on morphological criteria and/or morphometric analyses, often requiring the input of taxonomic experts. Unfortunately, there are too few taxonomic specialists available for the many research disciplines [9]. In addition, the enormity of biodiversity is often underestimated, and is continually threatened due to ongoing global change, therefore a comprehensive inventory appears to be an ever more urgent requirement [10]. Complicating the issue further, precise species identification based solely on morphological criteria can be extremely complex. Larval and/or immature stages can be morphologically very different from the imago or adult phase [3], [11], sexual dimorphism can be extreme [12], and cryptic and/or sister-species nearly identical [13]. Finally, identification based on morphology alone is often impossible either due to poorly preserved specimens, or to the difficulties associated with identifying non-invasive samples such as feces, bones in bird's pellets, shed skin, etc., but also to incomplete or degraded museum specimens. Similarly, illegally traded products from endangered species are often processed to such an extent that they are useless for forensic investigations based on morphological criteria [8] [14].

For these reasons, species identification via molecular methods, such as molecular barcoding using a short genetic marker [15], is proposed to overcome some of the weaknesses of the traditional morphology-based taxonomic system [16]. These newer methods will aid non-taxonomists by fulfilling the urgent requirement for rapid and accurate species identification tools [16]. In addition, providing that DNA can be adequately extracted and amplified, these methods have the advantage of using only a portion of the specimen or non-invasive sample for accurate species identification.

In theory, in order to accurately discriminate between closely related species, suitable molecular identification markers should exhibit low intra-species genetic variability, but high inter-species variability. Ideally, a single “universal” genetic marker should be used to facilitate the rapid identification of any living organism. To this end, the international project *Barcoding of Life* (www.barcoding.si.edu) aims to generate a complete species identification catalogue for all animal kingdom organisms based on the mitochondrial (*mt*) gene of the cytochrome *c* oxidase I (COI). Unfortunately, most of these COI sequences are until now inaccessible. However, sequences of another *mt* gene, cytochrome *b* (cyt*b*), are more abundant and freely available in public databases even if their quality is not always optimal [17]. This perhaps explains why this marker is most often used for species identification in vertebrates [18] and particularly for mammals [19].

The current protocol for molecular barcoding is based on PCR amplification of an *mt* marker, followed by “classical” Sanger sequencing. This robust approach is effective when applied to a few samples, but appears inefficient and expensive when scaled up to thousands of samples. Additional difficulties such as heteroplasmy (several *mt* genomes co-existing within the same cell [20]) or Numts (copies of *mt* DNA that are integrated into the nuclear genome, [21]–[23]) further frustrate the task of species identification. Similarly, DNA mixtures extracted from non-invasive samples (*e.g.* predator and prey DNA mixes from feces or bird's pellets; reviewed in [24]) also create problems for species identification without labor-intensive cloning.

Significant advances in high throughput Next Generation Sequencing (NGS) technology have allowed us to develop a novel barcoding method for the fast and accurate identification of wild rodent species. Such an innovative approach was recently proven effective in the correct species assignation of 255 insect specimens, corresponding to 17 different species of Ephemeroptera and Trichoptera [25]. We opted to utilize the 454 GS-FLX (Roche™) high-throughput sequencing system due to the following benefits. Firstly, read lengths are considerably longer at approximately 400 bp, compared to other NGS technologies (*e.g.* Illumina/HiSeq 2000, ∼100 bp; Life Technologies/SOLiD 3, ∼50 bp [26]) allowing complete sequencing reads for PCR products between 100 and 300 bp. To correctly identify an animal species, more than 100 bp are usually required, regardless of whether COI or cyt*b* is used [27], [28], while 200–250 bp corresponds to the maximum upper limit of markers able to target fragmented and/or degraded DNA [29], [30]. In addition, the high number of sequences produced (*i.e.* 1,200,000 sequences per run) combined with a suitable tagging method allows the concomitant identification of hundreds or thousands of samples in a single run [31]. As such a tagging method consists of appending an additional 50 to 60 bp to both ends of the targeted fragment (30 bp for Titanium adaptors, ∼10 bp for the tag sequence, ∼20 bp for the PCR primer), the total read length could reach 200 bp. Unlike the classical Sanger method, the 454 technology includes an emulsion PCR (emPCR) prior to the pyrosequencing step [32]. This step isolates each DNA strand before sequencing, mimicking sequencing via cloning. This method is thus extremely well-suited for the analysis of DNA mixtures, in which ambiguous heteroplasmy cases and misleading Numt amplifications could be resolved, and where prey and predator sequences could be easily unraveled when investigating feces or bird's pellets.

We chose to focus our study on rodent identification, as rodents represent 40% of all mammalian species [33]. Significant difficulties are currently associated with the correct identification of rodent species, due to the many cryptic species [13], [34], [35], and their ever increasing numbers, as new genera and species are continually described (*e.g. Laonastes aenigmamus*, [36]; *Saxatilomys paulinae*, [37]; *Mayermys germani*, [38]; *Tonkinomys daovantieni*, [39]). Furthermore, rodents are one of the preferred subject for epidemiology, agronomy and ecology investigations, not only due to their existence as major hosts and vectors of human parasites and pathogens (reviewed in [40]), but also as major agricultural pests. Rodent species identification is often difficult using morphological criteria alone [13], [34], [35], [41], while accurate identification is absolutely essential in such studies. Despite these difficulties, a clear picture of rodent taxonomy is nonetheless emerging, resulting in a reliable baseline reference against which a relevant molecular barcoding method can be developed.

In this study, we analyzed 946 rodent samples representing the breadth of rodent diversity, which included 820 tissue samples preserved in ethanol, 49 rodent feces, 54 rodent skins from museums, 12 feces from carnivores likely to have ingested rodents, and 11 bird's pellets containing bones of micro-mammals. Firstly, we designed a small DNA-barcode able to discriminate the largest number of rodent species. We then tested its efficacy on 265 reference samples corresponding to 103 rodent species. Finally, its applicability was successfully evaluated using delicate samples such as non-invasive and museum samples, demonstrating that this new method could open new realms of identification possibilities in many fields of biology.

## Results and Discussion

### Mini-barcode selection

We compared the efficiency of the two most common *mt* gene used to discriminate and identify rodent species: firstly, the Consortium for the Barcode of Life (CBOL) standard animal barcode, cytochrome *c* oxidase I (COI); and secondly, cytochrome *b* (cyt*b*), the marker most commonly used to investigate mammal biosystematics [19]. GenBank searches using the keyword ‘cyt*b*’ yielded 15,121 sequences corresponding to 1,476 rodent species, whereas ‘COI’ retrieved only 2,857 sequences corresponding to 503 rodent species. Furthermore, only 42% of these COI sequences were identified at the genus or species level, therefore the remaining sequences, labeled as ‘Rodentia sp.’, were not included for assessment. Only sequences of species available for both markers were selected and only one sequence per species was conserved. Sequences with ambiguous or incorrect species assignation were discarded, as well as incomplete or poor quality sequences (*i.e.* sequences with undetermined nucleic acids at numerous positions). As a result, a total of 115 rodent species sequences were available for both *mt* markers (see Fasta S1 for COI and Fasta S2 for cyt*b*). All Fasta alignments (Fasta S1, S2, S3, S4, S5, S6, S7, S8, S9, S10, and S11) were deposited in the Dryad data repository (http://datadryad.org/; doi: 10.5061/dryad.1j6v6).

Following [27], the predicted efficacy of successive 100 bp-long fragments for both markers were then assessed via three parameters: i) resolution percentage from neighbor-joining (NJ) analyses (%Res), ii) the mean pairwise genetic distance using the Kimura two-parameter substitution model (%K2P) and iii) mean variable sites percentage (%Var).

Both markers encompass several 100 bp mini-barcodes, which give 100% of resolution for the GenBank rodent dataset (Table 1). It means that using these mini-barcodes, all the 115 species of the GenBank dataset could be discriminated based on the NJ tree. Interestingly, some of these mini-barcodes were more variable than the entire gene (Table 1). For example, the cyt*b* fragment located between positions 901 and 1000 exhibited 78.0% variable sites, while the whole cyt*b* gene, 60.1%. This also held true for the COI marker, with 48.0%Var for mini-barcode 1–100, versus 43.1%Var for the entire gene. In general, cyt*b* barcodes were more variable than COI barcodes (19.9–41.4%K2P *versus* 20.2–25.5%K2P, and 46.0–78.0%Var *versus* 42.0–48.0%Var). Of all analyzed mini-barcodes, the cyt*b* barcode located between positions 701 to 800 appeared the most promising. In addition, it was surrounded by two regions of low variability (601–700 and 801–900), facilitating the design of rodent universal primers. Furthermore, owing to the fact that there are more cyt*b* rodent sequences available in GenBank than those for COI, permits the use of GenBank sequences as a reference database. Accordingly, the cyt*b* mini-barcode located between positions 701 to 800 was selected for all subsequent rodent species identification.

**Table 1:** Estimation of discrimination capability for the different 100 bp COI and cyt*b* mini-barcode genes on 115 rodent species.

	Length	% Res	% K2P	% Var
**COI**				
Standard size	647	100	22.4	43.1
1 to 100	100	100	25.5	48.0
101 to 200	100	100	21.2	39.0
201 to 300	100	98.3	20.2	43.0
301 to 400	100	98.3	22.5	42.0
401 to 500	100	96.5	21.5	42.0
501 to 600	100	96.5	25.4	44.0
**cyt** ***b***				
Standard size	1140	100	27.8	60.1
1 to 100	100	100	26.3	60.0
101 to 200	100	98.3	26.6	56.0
201 to 300	100	100	23.8	54.0
301 to 400	100	98.3	31.9	57.0
401 to 500	100	100	19.9	46.0
501 to 600	100	100	25.9	58.0
601 to 700	100	100	30.1	58.0
701 to 800 *	100	100	33.7	66.0
801 to 900	100	100	23.2	55.0
901 to 1000	100	98.3	41.4	78.0
1001 to 1100	100	98.3	27.9	63.0
**Cyt** ***b*** mini-barcode **				
666 to 801	136	100	34.9	66.2

Res, resolution in neighbor-joining analysis; K2P, genetic distances based on K2P nucleotide substitution model; Var, variable sites. The best 100 bp barcode to identify rodent species is indicated with * based on these three statistics. The mini-barcode designed in our study is indicated with ** and encompasses the best 100 bp barcode.

### Primer design

To design universal rodent primers we used the initial cyt*b* dataset extracted from GenBank (15,121 sequences), but removed poor quality or Numt sequences, resulting in 9,071 sequences, which corresponded to approximately 1,063 rodent species (Fasta S3).

Primers were designed to target the most conserved parts of the gene surrounding the selected mini-barcode. Several nucleotide positions (often at the third nucleotide) were degenerated in order to allow hybridization to multiple rodent species DNA templates. Due to primer design constraints, the final mini-barcode corresponded to a fragment slightly longer than 100 bp (136 bp), but still small enough to target fragmented/degraded DNA, located between positions 666 (L15411) to 801 (H15546) of the cyt*b* gene (Fasta S3).

The forward and reverse primers used are as follows: L15411F 5′-GAY AAA RTY CCV TTY CAY CC-3′ and H15546R 5′-AAR TAY CAY TCD GGY TTR AT-3′ respectively. To allow sample assignment, primers were modified into fusion tagged primers following [31]: a seven bp tag was added to each primer at its 5′ extremity, as well as a 30 bp adaptor for the 454 Titanium sequencing reagent series, resulting in a final amplicon size of 250 bp.

### PCR, 454 pyrosequencing and SESAME software analysis

The applicability of our molecular mini-barcode was investigated in a wide range of samples (Table S1). PCRs were performed on 820 ethanol-preserved tissues (265 reference samples and 555 non-identified samples), 49 rodent feces, 54 museum skins, 12 carnivore feces and 11 owl's pellets, all corresponding to at least 180 different rodent species. Forty-seven ethanol-preserved samples were randomly selected for duplicate analysis, while amplifications performed on non-invasive and museum samples were systematically duplicated. In order to estimate the 454 pyrosequencing error rate, two clonal *mt* fragments (plasmids containing PCR products) were amplified in 24 independent PCRs. Overall, 1,140 PCR attempts were realized: 1,093 yielded positive amplifications, 13 gave weak amplifications and 34 failed. Most of the PCR failures concerned samples with degraded DNA: museum skins (19 failures), owl's pellets (4) and feces from rodents (6) or carnivores (1).

Following 454 GS-FLX Titanium pyrosequencing, 197,650 reads were obtained. Sequences differing by at least one base pair substitution or by indels were called “variants” [31]. Among the resulting sequences, we distinguished “artefactual variants” (variants arising from PCR, emPCR or pyrosequencing errors) and “true variants” (variants that were retained following our validation procedure, see Methods), which will hereafter be called “haplotypes”. A total of 114,293 reads corresponding to 16,439 distinct variants were subsequently assigned to 1,103 samples via the forward and reverse tag combination using the software SESAME [42]. There were 104 mean reads per sample, although more than 20 reads were obtained for 98.4% of samples, and more than 50 for 89.1%. Artefactual variants were sorted and discarded manually based on the alignments generated for each sample in the SESAME software.

### Accuracy and quality assessment of the 454 reads

The 454 pyrosequencing technology is known to produce a significant proportion of sequencing errors, therefore it is necessary to be able to identify and discard these artefactual variants [31].

In order to accurately estimate the error rates, internal controls were included. They consist in 24 PCR products performed on clonal cytb fragment of two different rodent species, and whose genuine sequences were obtained by classical Sanger sequencing method. The 6,109 pyrosequencing reads were compared to the genuine sequences to calculate the percentage of erroneous reads, and to also validate our selection procedure and our ability to discriminate between artefactual variants and haplotypes.

The percentage of reads with at least one substitution (Sub), insertion (Ins) or deletion (Del) were estimated. Based on the 16 PCR amplicons obtained from a single clone (eight PCRs per clone), we determined that 65±1% and 64±7% (mean ± S.D.) of the 454 sequences were perfectly identical to the Sanger sequences. Errors rates were similar for both clones, (and also between the different PCRs from the same clone): Sub = 4±2% and 6±3%; Ins = 24±5% and 27±6%; Del = 6±2% and 3±1% for the two clones respectively.

To estimate the percentage of recombinant chimeric reads (Chim) the two clones were pooled together and eight independent PCRs were performed, resulting in 58±7% of 454 reads that were strictly identical to the Sanger sequences, with the remaining error rates: Sub = 4±2%; Ins = 23±5%; Del = 7±3%. Chimeras were easily identified as spurious sequences derived from the mixed clonal template, with an estimated 8±4% chimeric reads.

As expected [43], the most common sequencing errors were insertions, likely due to homopolymer stretches, and which were detected in more than 20% of the reads generated for our internal controls. Nevertheless, even if large numbers of artefactual variants were generated, they were easily identified and removed from the analysis due to their frame-shifting effect on the coding sequence.

Clonal variants detected at the highest frequency during 454 pyrosequencing were selected as the true variants, which always corresponded to the genuine sequences obtained via Sanger sequencing. Consequently, we applied the same rationale to determine the haplotype for subsequent rodent samples. Confirmation was obtained when 46 tissue samples were processed in duplicate, with subsequent independent haplotype selection yielding exactly the same haplotype. Altogether these findings demonstrate that this protocol ensures accurate haplotype selection. The validation procedure was slightly different when analyzing non-invasive samples (see Methods), as extracted DNA likely corresponded to several different organisms.

### DNA barcoding of reference samples

The ability of our mini-barcode to identify rodents at the species level was first tested on a reference sample comprising 265 rodent individuals corresponding to 103 species from Asia, Africa and Europe (Table S1 and Fasta S4). The exact species of each sample had previously been clearly established using molecular and/or morphological techniques. Representatives of several rodent genera known to be difficult to discriminate at a species level using morphological characteristics alone, such as *Mastomys* [34], [44], *Microtus* [45] or *Gerbillus* [13], were included in the reference dataset, as well as recently diverged species such as members of the *Rattus rattus* complex [46].

**Figure 1:**
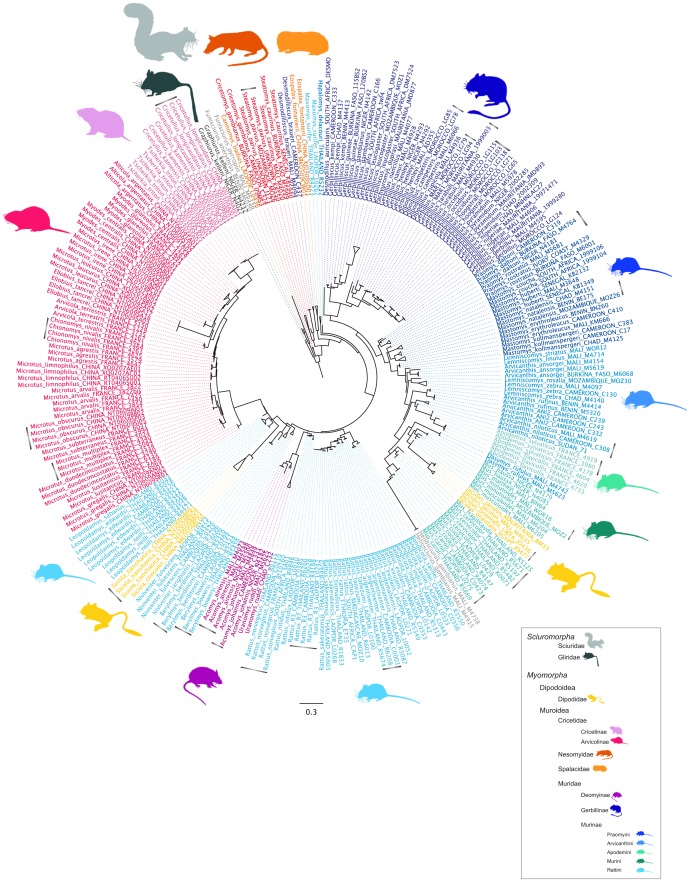
ML tree obtained from the analysis of the 136 bp mini-barcode (cyt*b*) on the rodent reference sample (265 individuals, 103 species). Gray bars indicate terminal nodes with indicated bootstrap values (Bp) <80%.

In the maximum likelihood (ML) phylogenetic tree obtained from the analysis of the 136 bp barcode (Figure 1), representatives of the same species constituted monophyletic groups, which were supported by high bootstrap values (Bp>80%). Even closely related species were clearly distinguished (see for example the distinction between species of the genus *Rattus*, *Myodes*, *Microtus* and *Gerbillus*). A few species were however poorly identified as monophyletic (Bp<80) and were thus not distinguishable from their sister species. In all cases, these results either corresponded to groups whose taxonomy has not been officially confirmed, for example, the species status of *Microtus obscurus* in China, *Acomys johannis* in Mali, or *Gerbillus* representatives in Morocco remain controversial [47], [48]; or to species which have recently diverged, such as *Microtus arvalis* versus *M*. *obscurus*.

**Figure 2:**
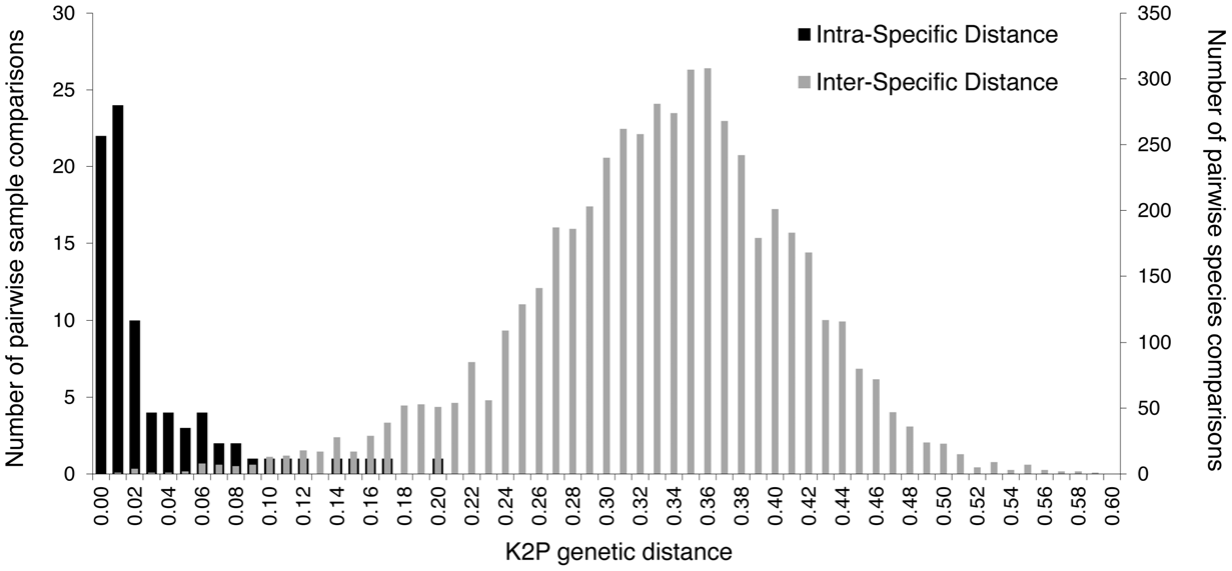
Distribution of pairwise K2P genetic distances within and between 103 rodent species (265 individuals) based on the 136 bp mini-barcode (cyt*b*).

The comparison of pairwise K2P genetic distances within and between species shows a gap centered on 10% (Figure 2). The mean intra-specific distance reached 2.8% (S.D. 4.3%) while the mean inter-specific distance was 32.7% (S.D. 8.1%). In accordance with the ML tree, some closely related species or sibling species displayed very low K2P distances. This was the case for *Microtus arvalis* and *M*. *obscurus*, which diverged at only one or two nucleotides within our mini-barcode (K2P = 0.9%). However *M*. *obscurus* is sometimes considered as an isolated lineage of *M*. *arvalis* rather than as a valid independent species [49]. Similarly, *Rattus sakeratensis*, *R.* lineage R3 and *R. tanezumi* exhibit small distances (1.8–5.4%) corresponding to between two to seven substitutions. These findings are in accordance with recent phylogenetic studies which estimate that they likely diverged less than one million years ago [46], questioning the distinct species status of *Rattus* R3 [41]. Indeed, if speciation events had occurred recently, not enough time has elapsed for mutations to become fixed within the species. Consequently, whichever small DNA marker is considered, it would be extremely difficult to discriminate between very closely related species.

Accidental amplification of Numts occurred in most species from the genera *Arvicola*, *Apodemus*, *Gerbillus*, *Microtus*, *Myodes* and *Praomys* similar to that reported in the literature (*Microtus*, [50], Arvicolinae, [51], *Myodes gapperi*, [52], *Apodemus sylvaticus*, [23]). In these cases, Numts did not hinder species identification because: i) they were amplified at a lower frequency than the multicopy *mt* marker and ii) they were easily identified as Numts owing to the large number of *Apodemus* and *Microtus* species sequences deposited in GenBank.

However, if Numts were only recently incorporated into the nuclear genome, frameshift mutations are unlikely to have become fixed. Recent Numts could be problematic if not documented in the literature, and could become difficult to distinguish from genuine cyt*b* sequences [53]. Nonetheless, in such cases, Numts are generally species specific, and could therefore also potentially be used as species markers to confirm identification obtained with the *mt* mini-barcode [54].

### DNA barcoding of unknown samples

To experimentally validate our mini-barcode, we aimed to identify the species of 555 samples, for which there was no previous identification (Table 1 and Fasta S5). PCR amplification failed for only three samples. Depositing these three DNAs on an agarose gel suggested that the DNA concentration for these samples was very high and probably inhibited the PCR reaction. Of these 555 samples, 85.6% were identified using the BLASTN program and GenBank rodent reference sequences (Table S1). The mean number of substitutions within species was estimated to 3.5% from our reference rodent dataset. Consequently, to assign a species to an input sample sequence, we fixed a threshold of 96% BLAST identity (with 99–100% of coverage).

The success of species identification was highly variable. It depended on the amount of knowledge available on local rodent fauna, which in turn differed according to the various geographic areas that were investigated. For example, the identification success rate was 100% for European samples (N = 104, Maximal Identity: Max Ident ≥99%). The taxonomy of the European rodent fauna is today well known and largely documented (*e.g.* [55]), numerous molecular studies have been performed on European species (*e.g.* [45], [56], [57]) and sequences have been deposited in GenBank. Similarly, rodents from the Indochinese region (N = 269) have recently been subjected to intensive phylogenetic studies [41], [46], [58]–[62], http://www.ceropath.org/ as also performed for West African rodents (N = 64) [44], [63]–[67]. Consequently rodent samples from these areas displayed high identification success rates (Indochinese samples = 94.7%, with Max Ident ≥98%, rates for West Africa = 100% with a Max Ident ≥99%). In contrast, samples from the Sundaic region (N = 21) and from East Africa (N = 95), where rodent faunas have remained relatively unexplored, resulted in lower identification success rates (61.9% and 40.0% respectively, with Max Ident ≥96%). These observations underline the absolute necessity of a robust reference dataset including accurate taxonomic differentiation, for this barcoding approach to be applicable.

### DNA barcoding of degraded rodent samples

DNA extracted from non-invasive samples is often low in quantity and of poor quality [68]. It is thus subject to contamination by exogenous DNA and can often be co-extracted with Taq polymerase inhibitors [68], [69]. Problems encountered when working on museum specimens are similar to those faced when dealing with non-invasive samples, but are clearly exacerbated [70]. Post-mortem DNA degradation (*e.g.* depurination, deamination) is known to lead to artefactual substitutions during PCR [71], [72]. Researchers working on degraded DNA often circumvented this problem by cloning and sequencing independent PCR amplicons [70], a procedure which is time-consuming and expensive. Consequently, our rodent species identification method appears to be well suited for degraded substrates since it targets a small DNA barcode (136 bp), and avoids a long cloning process. To prove this hypothesis, our rodent species identification method was thus evaluated on rodent feces or museum samples.

#### Rodent feces

Trapping rodents requires complicated logistics (trap maintenance and transport), and staff specially trained in rodent handling. These requirements are often difficult to meet during field trips. In addition, some rodent species are elusive or are resistant to trapping, despite local abundance (*e.g. Arvicanthis ansorgei*, *Gerbillus henleyi*, *Rattus norvegicus* [73]) or are endangered protected species (*e.g. Laonastes aenigmamus* [36]). In such situations, collecting non-invasive samples such as feces appears as an attractive alternative. Our approach was tested on 49 rodent feces collected in traps from West Africa (N = 11) and Asia (N = 38) (Table S1). In this fashion, it was possible to compare sequences obtained from both the feces and tissue from the same animal. In addition, amplifications were performed twice per faecal samples. Indeed, under an extreme scenario, the amplification could start from a single template molecule because of the low DNA quantity of the faecal extract. If this template was chemically modified by DNA decays, artefactual mutations due to nucleotidic misincorporations during the PCR reaction could lead to an inaccurate sequence.

Of the 49 fecal samples, duplicate PCRs failed on three samples, and for another provided a spurious avian haplotype, however 45 fecal samples had identical replicate results, and sequences were indistinguishable from those obtained from the animal's corresponding tissue. Anecdotally, some feces samples from black rats in Mali gave additional divergent haplotypes corresponding to birds (Table S1, and Fasta S6). Black rats are known to be an opportunistic species, reported to feed on small animals and carrion, neatly corresponding with our findings.

#### Rodent museum samples

Taxonomic investigations require repeated comparisons between modern and museum specimens (often the species holotype). Such comparisons could lead to inconclusive identification when the holotype is poorly preserved, or when intra-species variation is so large that the holotype appears morphologically distinct from the novel specimen requiring identification. For example, the wide range of intra-species morphological variation makes such criteria unsuitable for accurate rat identification, resulting in over-described species with confusing taxonomy, hampered by an overabundance of synonyms. In this case, sequences obtained from museum holotype specimens have been used to refine the taxonomy of the tribe Rattini [41]. However, such holotype specimens are extremely precious. Therefore, for our preliminary tests, our identification method was applied using less valuable museum specimens.

We tested our approach on 54 DNA extractions kindly provided by the Museum National d'Histoire Naturelle (MNHN) of Paris. These DNAs had been extracted in 2006 from museum samples prepared between 1958 and 1991. Based on morphological characteristics, they were assigned to five genera (*i.e. Hybomys*, *Hylomyscus*, *Lemniscomys*, *Lophuromys* and *Praomys*), however, their actual species remained uncertain (Table S2). Previous attempts of 500–800 bp *mt* amplification had failed for 24 samples (V. Nicolas pers. comm.). However, using our method, 49 of the 54 DNA samples were amplified at least once (90.7%) while 40 were amplified twice (74.1%). For 41 of the positive samples, the highest frequency variant obtained was assigned to one of the five expected genera (Fasta S7). They represented 6,243 reads from 7,482 validated reads (*i.e.* 83.4%). For six samples, the species result differed from the expected identification (Max Ident = 100% with *Mus setulosus*, *Malacomys edwardsi* and *Mastomys natalensis* - 11.0% of the true variants - see Table S2 and Fasta S7 for details). These three species corresponded to taxa currently housed in the museum, therefore the discrepancies could be due to museum labeling error. However, these DNA samples had never previously yielded positive PCR amplifications. As potential higher quality contaminants can be preferentially amplified when working with degraded templates, our results are likely due to contamination by exogenous rodent DNA during the extraction procedure. Sequences obtained for the remaining two positive samples were identified as human (Table S2 and Fasta S8) and were definitely due to contamination.

In addition to the most abundant haplotype, several other haplotypes were also validated for a high proportion of the museum samples (83.3% of the sampling harbour between 2 to 11 haplotypes, with a mean average of three haplotypes per sample, Table S2 and Fasta S9). These ‘additional’ haplotypes represented 5.6% of the validated reads, of which 1.8% corresponded to African rodent genera not included in our study, and which are so completely morphologically different, it is unlikely they could have been confused with the museum specimens in question. In addition, 1.8% of reads corresponded to other non-African rodents, and also to shrews, bats, ungulates, primates, carnivores, birds and reptiles. The remaining 2.0% corresponded to human haplotypes/Numts. These haplotypes were found at very low frequencies in most of the museum samples investigated. Consequently, plentiful evidence exists to suggest contamination during the DNA extraction process.

### Environmental barcoding: molecular diet analysis of micro-mammal predators

We assessed the suitability of our approach in determining the diet of rodent predators, as rodents are an important link in the food chain for many wild species [74]. Species identification of rodent remains in carnivore feces and bird's pellets is thus a central issue to ecological studies on food chains and webs, prey-predator relationships or competition between predators. A potentially innovative application could be the use of owl pellets in enabling a faunal census of those elusive or difficult to trap rodents [75], [76].

We analyzed 12 carnivore feces (mustelids, felids and canids) and 11 owl's pellets, all collected in France. Based on external characteristics, feces were *a priori* identified as those of wild cats (*Felis silvestris*, n = 4), pine martens (*Martes martes*, n = 4) and red foxes (*Vulpes vulpes*, n = 4). Four samples were identified as barn owl pellets (*Tyto alba*) and seven remained undetermined. All of these species are known to prey on rodents. From 23 PCRs, 20 resulted in replicated positive amplifications. One positive result could not be replicated while two owl's pellets never yielded positive amplification (Table 2). With respect to feces samples, 88.7% of the 1,205 validated reads were assigned to the defecator, 7.5% corresponded to ingested rodents (Fasta S6 for prey and Fasta S10 for predators). On the contrary, for bird's pellets, 95.4% of the 1,481 validated reads could be identified as rodents while only 0.3% as owls (the barn owl, *Tyto alba* and the long-eared owl, *Asio otus*). For both feces and pellets, we observed excellent result repeatability in the two replicates for each sample: identifications were obtained in 78.6% and 77.3% of both replicates for feces and pellets respectively. Molecular identification confirmed the preliminary carnivore species identification based on external sample characteristics, except for two samples: feces supposed to have originated from pine martens were revealed to be those of red foxes.

**Table 2:** Predator and prey haplotype occurrence in both carnivore feces and bird's pellets.

		Feces												Bird's pellet								
		Cat				Pine marten				Red fox				Barn owl			owl					
Order	Common name (Scientific name)	1	2	3	4	5	6	7	8	9	10	11	12	14	15	16	17	19	20	21	23	24
**Predator:**																						
Carnivora	Wildcat or domestic cat (*Felis silvestris* or *F. catus*)	75/59	0/15	88/91	49/36																	
	Pine marten (*Martes martes*)					188/169			45/57													
	Red fox (*Vulpes vulpes* - 99%)						4/1	45/50		15/1	9/4	11/23	23/11									
Strigiformes	Long-eared owl (*Asio otus*)																			3/1		
	Barn owl (*Tyto alba*)																					1/0
**Ingested species:**																						
Rodentia	Water vole (*Arvicola scherman* - 99%)	1/0		0/3	9/14			3/1									22/13	57/70	123/229	108/128		
	Fiels vole (*Microtus agrestis*)														0/8						82/147	
	Common vole (*Microtus arvalis*)			7/3	5/4			0/1			3/1				17/16	89/87	41/39	2/9				
	Pine vole (*Microtus subteraneus*)														13/2							
	Bank vole (*Myodes glareolus*)					12/9						8/4										
	Numts from vole (Arvicolinae)					*2/0*										*1/0*			*4/9*	*2/4*	*10/3*	
	Wood mouse (*Apodemus sylvaticus*)													22/18								2/7
	Numts from wood mouse (*Apodemus sylvaticus*)													*12/4*								*6/2*
	Harvest mouse (*Micromys minutus*)																					0/5
Soricomorpha	Crowned shrew (*Sorex coronatus*)								3/3						20/8					5/11	2/2	
	Greater white-toothed shrew (*Crocidura russula* - 99%)																				1/0	
Passeriformes	Tree pipit (*Anthus trivialis*)							8/2														
	Common blackbird (*Turdus merula*)								4/12													
Artiodactyla	Wild boar (*Sus scrofa* - 99%)											0/1										
Haplotaxida	Earthworm *(Lumbricus terrestris* - 96%)								8/2		1/0											
**Environmental contamination:**																						
Proteobacteria	*Caulobacter* sp (86%)													1/2								
	*Pseudoxanthomonas spadix* (85%)														0/2							
	*Stenotrophomonas maltophilia* (94%)										1/0											
Primates	Human (*Homo sapiens*)								0/1													
unidentified	No match													0/8								0/1

The number of sequences is indicated for each replicate PCR1/PCR2. Blast results are indicated in parentheses only when Max Ident <100%. Numt sequences are italicized. PCR attempts failed for one out of the two replicates performed for sample 2. Both replicates failed for samples 13 and 22. Feces samples 6 and 7 thought to have originated from the pine marten were revealed to be those of the red fox.

Based on 454 sequencing, wild cats were demonstrated to ingest water voles (*Arvicola scherman*) and common voles (*Microtus arvalis*). The pine marten's diet was composed of bank voles (*Myodes glareolus*) but also of non-rodent species such as the crowned shrew (*Sorex coronatus*), the common blackbird (*Turdus merula*) and the earthworm (*Lumbricus terrestris*). As blackbirds and earthworms were both found in one single feces sample, they could represent a case of secondary predation (*i.e.* an earthworm ingested by a blackbird, which was in turn ingested by a marten) [77]. Red fox feces included three rodent species (*A. scherman*, *M. arvalis* et *M. glareolus*) but also an avian species such as the tree pipit (*Anthus trivalis*) and earthworms (*Lumbricus terrestris*). Anecdotally, a single read of wild board (*Sus scrofa*) was detected in feces from a red fox and could correspond to scavenging. Proteobacteria (*Stenotrophomonas maltophilia* with Max Ident = 94%) was also identified in a red fox feces sample (Fasta S11).

Owl's pellets contained sequences of four arvicoline rodents (*A. scherman*, *M. arvalis*, *M. agrestris* and *M. subterraneus*), two murine rodents (*Apodemus sylvaticus* and *Micromys minutus*), and two shrews (*Sorex coronatus* and *Crocidura russula*). Two proteobacerial sequences close to *Caulobacter sp.* (Max Ident = 86%) and *Pseudoxanthomonas spadix* (Max Ident = 85%) were also identified.

## Conclusions and Perspectives

Our study reports the successful design of a 136 bp cyt*b* mini-barcode which accurately assigns individual rodent species. The applicability of our mini-barcode for species identification relied on its ability to discriminate between intra-species and inter-species levels despite its short length. As shown by the ML tree including 265 reference samples (103 species), intra-species clades are easily distinguished (Bp>80%) and appear clearly distinct from closely related species. Mean inter-species genetic distance (K2P = 32.7±8.1%) was higher by one order of magnitude than the mean intra-species distance (2.8±4.3%). Exceptions to this rule mainly concerned groups whose taxonomy is still unclear, or experiencing recent or ongoing speciation (*e.g. Microtus* spp., *Gerbillus* spp., *Rattus* spp.).

Altogether, hundreds of ethanol-preserved samples representing more than 180 different species from Europe, Asia and Africa were amplified, with a very low failure rate (only three samples - 0.4%). Assignation of rodent species using BLASTN and GenBank reference sequences with a stringent criterion (Max Ident ≥96%), was achieved for 85.6% of samples. However the assignation success rate depended on the availability of both local fauna taxonomic knowledge, and publicly accessible molecular data, which is illustrated by the contrasting results for samples originating from Europe, Indochina or West Africa compared to those issuing from the Sunda or East Africa). In addition, exact assignation depended upon the accuracy of the taxonomic identification associated with GenBank sequences.

The combination of a mini-barcode, 454 pyrosequencing technology and the tagging method developed in [31] allowed reliable, fast and inexpensive species identification for a large set of samples in one quarter of a run (1,140 samples multiplexed in this study). This protocol results in a mean coverage of 104× per sample. However, coverage for good quality DNA samples could probably be reduced to 20× without altering the taxonomic assignation rate. Consequently, up to 5,000 samples could be identified using our method, considerably decreasing the costs of molecular identification per sample for large-scale studies (less than three euros for 1,140 samples; less than one euro for 5,000 samples). In addition, a novel inexpensive NGS method, Ion Torrent technology from Life Technologies, has recently produced reads well over 200 nucleotides long with error rates similar to those observed in 454 runs. In future, our procedures could be combined with this novel sequencer, considerably reducing the cost of individual identification.

This barcoding approach relies on the clonal sequencing of a short multicopy DNA fragment and thus appears suitable for studies based on low quantity degraded or fragmented DNA. Encouraging results were obtained with feces samples, such that species identification was achieved for 94% of the samples, and comparison with sequences obtained from fresh material collected from the same rodent demonstrated 100% identity. Results obtained on museum samples were also fruitful despite the lack of stringent ancient DNA extraction procedures. Genuine sequences were obtained even if contaminating sequences were also detected. These results reveal the high sensitivity of the method when dealing with scarce DNA.

In addition, novel degenerated primers combined with non-stringent PCR conditions and clonal sequencing, facilitated the investigation of mixed DNA samples. Other molecular identification methods such as DNA arrays or those based on species-specific primers require a certain anticipation of the result. Micro- and macro-arrays rely on the hybridization of short specific nucleotide probes to target organism DNA with subsequent detection of the hybridization signal [18]. Consequently, these methods require prior knowledge of which different species could potentially be encountered, and therefore cannot detect unexpected, unknown or newly described species [27]. Moreover, due to reduced genetic similarity, undiscovered haplotypes or geographic variants would fail to properly anneal to the array probes. Species-specific primers are usually designed for a limited species set. An unknown species could mimic the pattern expected for a well-known species and once again, would not be correctly identified [34]. Our method is able to overcome the vast majority of these problems.

Finally, identification performed on carnivore feces and owl's pellets highlighted the enormous potential of our approach for use in ecological studies. Identified rodent sequences were consistent with typical prey species ingested by the carnivores and owls in the sampled area. Based on these preliminary results, it is still difficult to confirm if all prey species present in the feces or pellets were actually detected. However, results obtained from museum specimens tend to indicate that even DNA present in tiny amounts (such as contaminations) are likely to be detected with our method, but has yet to be confirmed in animal diet investigations. In future assays, it is likely that increasing sequence coverage and numbers of PCR replicates will enhance the probability of detecting less frequent prey. Our experiments also suggest previously unsuspected applications of our method in the field of ecology. A significant number of sequences were assigned to animals other than rodents, such as mammals, birds, reptiles but also invertebrates. Consequently our method could be suited to establishing the diet of animals which feed on prey other than rodents, as well as determining which predator species had produced the feces or pellet sample. Our approach was proven to be successful in determining unique carnivore species via their scat (cats, foxes or martens). In two cases, species assignments previously attributed by assessing the external aspect of the feces were proved erroneous and were subsequently corrected (marten *vs*. fox). Determining the owl species was less effective, probably due to the cleaning method performed before DNA extraction and which favors the selection of bones in the pellet. Further studies testing our approach with controlled samples, such as feces and pellets originating from captive animals with a pre-determined diet, or feces and pellets with previously identified contents via standard morphological approaches, would be of excellent use in refining this method.

## Materials and Methods

### Mini-barcode selection

Available rodent sequences for both COI and cyt*b* markers were extracted from GenBank, with only one sequence per species selected. Sequences were aligned by eye using BioEdit [78] and then partitioned into 100 bp mini-barcodes from the 5′ extremity of the gene.

For each marker and mini barcode, the NJ resolution percentage (%Res), mean pairwise genetic distance with the K2P model of substitution (%K2P) and the variable sites mean percentage (%Var) were all independently. To calculate the %Res, NJ trees were constructed with the K2P model of substitution and uniform rate of variation among sites. 1,000 bootstrap replicates were performed. All positions containing missing data were eliminated (complete deletion option). %K2P were computed with the same options. All the evolutionary analyses were conducted in MEGA5 [79].

### Sample selection

Four types of samples were selected in this study. Firstly, 265 high quality tissue samples preserved in ethanol were used as references. They had been collected in Asia, Africa and Europe from rodents that were unambiguously identified at the species level by specialists, based on either morphological characters or molecular data (www.ceropath.org; www.bdrss.ird.fr/bdrsspub_form.php; [55]). Specimens were selected in order to maximize the number of species and various geographic locations. The total reference sample comprised 103 species, 38 genera and 8 families. In addition, this reference set included closely related species and cryptic species that were only recently described (*e.g.* species of the *Rattus rattus* complex, *Microtus* complex, *Gerbillus* complex, etc.).

Secondly, 555 samples preserved in ethanol but with uncertain taxonomic status were selected. Feces found in the traps were collected at the same time in order to compare results obtained using high quality DNA (from tissue) or poor quality DNA (from non-invasive samples). Tissue and fecal samples were obtained for 11 and 38 rodents from Mali and Thailand respectively.

Thirdly, 54 DNAs were extracted from museum specimens (skins) and kindly provided by the MNHN of Paris.

And finally, feces from predators that were likely to have ingested rodents were collected. Feces thought to have originated from four foxes (*Vulpes vulpes*), four martens (*Martes martes*) and four wild cats (*Felis silvestris*), as well as 11 owl pellets were analyzed to determine their rodent diets. Sample information is detailed in Table S1.

### DNA extraction

DNA from the 820 tissue samples preserved in ethanol was extracted using the DNeasy Tissue Kit (Qiagen) following the manufacturer's recommendations. Non-invasive samples (feces and bird's pellets) were handled in a different area of the laboratory to prevent contamination from high quality DNA samples. DNA was extracted using the QIAamp DNA Stool Kit (Qiagen), following the protocol designed for the isolation of DNA from human stools.

DNA extraction from museum specimens was attempted between 2006 and 2009 in the MNHN laboratory using the CTAB protocol [80]. However, unfortunately, it was not achieved following the ancient DNA standards [81].

### Primer design and PCR optimization

Alignments of 9,071 cyt*b* GenBank sequences corresponding to 1,063 rodent species were performed with BioEdit 7.0.9 [78]. Rodent universal primers were designed in order to amplify the cyt*b* fragment identified as the best mini-barcode for rodent species identification.

Following our recent tagging and multiplexing method for 454 pyrosequencing [31], primers were modified in-fusion tagged primers by adding a short 7 bp sequence (the tag) and 30 bp Titanium adaptors to the 5′ ends of L15411F (5′-CCATCTCATCCCTGCGTGTCTCCGACTCAGNNNNNNNGAYAAARTYCCVTTYCAYCC-3′) and H15546R (5′-CCTATCCCCTGTGTGCCTTGGCAGTCTCAGNNNNNNNAARTAYCAYTCDGGYTTRAT-3′). These adaptors were required for the emPCR and 454 GS-FLX pyrosequencing using Lib-L Titanium Series reagents. Each tag differed from the others by at least three substitutions to avoid misassignment of samples (see Table S1). We designed 36 and 32 different tags for the forward and the reverse primers, respectively. This allowed the generation of 1,152 putative unique combinations of forward and reverse tags and thus the ability to tag 1,152 different amplicons. Samples were processed in 96-well plates and cyt*b* amplicons were individually tagged according to these primer combinations, as described in [31].

PCR amplifications using DNA extracted from tissue or non-invasive/museum samples were performed in independent facilities and at different times. PCRs were carried out in a 10 µL reaction volume using 5 µL of 2× QIAGEN Multiplex Kit Buffer (Qiagen) and 0,5 µM of each primer. One µL of tissue sample DNA (*i.e.* approximately 30 ng) or 2 µL of feces, pellets or museum sample DNA, was added to each well. The PCR started by an initial denaturation step of 95°C for 15 min, followed by 40 cycles of denaturation at 94°C for 30 s, annealing at 45°C for 45 s and extension at 72°C for 30 s followed by a final extension step at 72°C for 10 min. PCR amplifications from feces, pellets or museum DNA were performed in duplicate. To ensure method reproducibility, 47 tissue samples were also analyzed in duplicate.

PCR, emPCR and pyrosequencing-induced substitution and indel errors were assessed by comparing to two internal controls (*i.e.* clones of known sequences). These controls corresponded to classical Sanger sequencing of purified cyt*b* clonal sequences from *Rattus argentiventer* and *Mus cervicolor* samples (accession number HM217362 and JQ685755 respectively). Using a clonal sequence as an internal control ensured that any differences observed between pyrosequencing reads and the reference sequence were likely to have been generated during the 454 process (Numt co-amplification, artefactual mutations due to DNA chemical degradations, or cyt*b* amplification in a heteroplasmic individual could be discarded). To estimate precisely the error rate, both clones were independently amplified eight times. In addition, to assess chimera production rates, the two clones were pooled together and eight independent PCRs were performed on the mix. Consequently, chimeras were easily identified as spurious sequences derived from the double clone templates.

### Amplicon pooling and 454 GS-FLX Titanium pyrosequencing

PCR products (3 µL) were verified on 1.5% agarose gels and the positive reactions were pooled in equal proportions. An initial mix was generated for each PCR plate: 4 µL of efficiently amplified PCR products or 7 µL of less efficiently amplified products were pooled together. These mixes were once again verified on 1.5% agarose gels prior to generating a final mix to obtain a single “super-pool”. To achieve this, 10 µL per pooled PCR from tissues samples, and 20 µL per pooled PCR from non-invasive or museum samples were mixed together.

The “super-pool” was then processed by Beckman Coulter Genomics (Danvers, Massachusetts). To eliminate putative non-specific PCR products, the pool was run on a microfluidic electrophoresis Pippin Prep (Sage Science) and fragments of the expected 250 bp size were selected. Following emPCR, amplicons were sequenced on a 454 Genome Sequencer FLX (Roche) in one quarter of a Titanium picotiter plate.

### SESAME software analysis

The SESAME software (SEquence Sorter & AMplicon Explorer) [42] ver. 1.1B was used to sort the sequences (*i.e.* individual assignment and removal of artefactual variants due to sequencing errors during PCR, emPCR and 454 sequencing). Utilizing the tag combinations, sequences were assigned to the sample from which the PCR amplicon was obtained. When PCR was performed on rodent tissue or fecal samples, generally one single high frequency variant was detected and was consequently considered to be the valid sequence. Other variants were also found at very low frequencies and were considered as artefactual reads generated during PCR, emPCR and pyrosequecing steps (see [43], [82] for a details). This rationale was corroborated by results obtained with the internal controls. Occasionally, when PCR was performed on tissue samples, additional medium-high frequency variants were detected. In these cases, Numt amplification was suspected, and was periodically validated by comparing to GenBank Numt sequences.

When PCR was performed on fecal samples from predators or bird's pellets, divergent variants were detected, therefore the variant found at the highest frequency among each of these clusters was selected as the true variant. Similarly, several rodent species were often ingested by predators, and several haplotypes were expected to be generated via our molecular identification method. Furthermore, when PCR was performed on museum samples, the same rationale was applied: several divergent variants were found and were selected for subsequent identification steps. Indeed, contaminations by exogenous DNAs were expected, since an appropriate ancient DNA extraction standard procedure was not used.

Each selected variant was then compared to sequences available in GenBank, EMBL, DDBJ and PDB (nr database) using the NCBI BLASTN program [83]. When the maximal identity (Max Ident) reached 96% (with 99–100% of coverage), the best GenBank match species was selected as the species identification (see Results for justification of the threshold).

Concomitantly to the publication of this study, an improved version of the SESAME software called |SE|S|AM|E| BARCODE was released [84]. This new automated procedure for species identification building a reference library (*e.g.* GenBank) should considerably facilitate this task.

### DNA barcoding of rodent reference samples

A reference sample comprising 265 rodent individuals from 103 rodent species were used to generate 265 mini-barcode sequences. To assess the reliability of the mini-barcode to discriminate between closely related rodent species, a Maximum Likelihood analysis was performed on this reference dataset using RAxML 7.0.4 [85]. As model choice is limited in RAxML, the general time-reversible (GTR) + Γ model (option –m GTRGAMMA) was selected for the cyt*b* dataset [86], [87]. Tree robustness was assessed using the rapid bootstrap procedure (option –f a) with 1,000 replications (option -# numberOfRuns) [88].

Intra and inter-specific pairwise genetic distances were determined using the Kimura two-parameter (K2P) substitution model [89] with MEGA5 [79].

## Supporting Information

Table S1 **Information about the study samples used.** “Specimen identification” corresponds to the prior species identification based on morphological data and/or molecular tools for the reference samples. Other specimens were identified based on external criteria. The suffix “bis” in *Individual code* indicates replicates. BLAST statistics are italicized when Max Identity of the mini-barcode with the sequences available in GenBank <96% (the species corresponding to the query was not documented in GenBank).
(XLS)

Table S2 **Frequencies of genuine rodent haplotypes and exogenous contaminations obtained for museum skin samples.** * represents PCR failure. The suffix “bis” in *Individual code* indicates replicates. Haplotypes with highest frequencies are highlighted in bold. Numt sequences are italicized.
(XLS)

Fasta S1 **Alignment of COI sequences from 115 rodent species from GenBank.**
(FAS)

Fasta S2 **Alignment of cyt** ***b*** **sequences from 115 rodent species from GenBank.**
(FAS)

Fasta S3 **Alignment of primers designed for the 136 bp mini-barcode amplification.** Including 9,071 cyt*b* sequences extracted from GenBank and corresponding to 1,063 rodent species.
(FASTA)

Fasta S4 **Alignment of rodent haplotypes obtained from the 265 reference samples.**
(FAS)

Fasta S5 **Alignment of the rodent haplotypes obtained from ethanol preserved samples.**
(FAS)

Fasta S6 **Alignment of the prey haplotypes obtained from fecal and pellet samples.**
(FAS)

Fasta S7 **Alignment of the rodent haplotypes obtained from museum samples.**
(FAS)

Fasta S8 **Alignment of the human haplotypes retrieved from rodent museum sample analysis.**
(FAS)

Fasta S9 **Alignment of the vertebrate haplotypes corresponding to contaminations detected in rodent museum samples.**
(FAS)

Fasta S10 **Alignment of the predator haplotypes detected in fecal and pellet samples.**
(FAS)

Fasta S11 **Alignment of the bacterial and unknown haplotypes detected in fecal and pellet samples.**
(FAS)

## References

[1] Balakrishnan R (2005) Species concepts, species boundaries and species identification: a view from the tropics. Syst Biol 54: 689–693. 16126664 10.1080/10635150590950308

[2] Hey J (2009) Why should we care about species? Nature Education 2.

[3] Lefort MC, Boyer S, Worner SP, Armstrong K (2011) Noninvasive molecular methods to identify live scarab larvae: an example of sympatric pest and nonpest species in New Zealand. Mol Ecol Resour 12: 389–395. 22189059 10.1111/j.1755-0998.2011.03103.x

[4] Nagoshi RN, Brambila J, Meagher RL (2011) Use of DNA barcodes to identify invasive armyworm *spodoptera* species in Florida. J Insect Sci 11: 154. 22239735 10.1673/031.011.15401 PMC3391933

[5] Lowenstein JH, Burger J, Jeitner CW, Amato G, Kolokotronis SO, et al (2010) DNA barcodes reveal species-specific mercury levels in tuna sushi that pose a health risk to consumers. Biol Lett 6: 692–695. 20410032 10.1098/rsbl.2010.0156 PMC2936149

[6] DeSalle R, Amato G (2004) The expansion of conservation genetics. Nat Rev Genet 5: 702–712. 15372093 10.1038/nrg1425

[7] Rosentreter R (2004) Sagebrush identification, ecology, and palatability relative to Sage-Grouse. USDA Forest Service Proceedings 1–14.

[8] Palumbi SR, Cipriano F (1998) Species identification using genetic tools: the value of nuclear and mitochondrial gene sequences in whale conservation. J Hered 89: 459–464. 9768497 10.1093/jhered/89.5.459

[9] Bacher S (2012) Still not enough taxonomists: reply to Joppa et al. Trends Ecol Evol 27: 65–66. 22138045 10.1016/j.tree.2011.11.003

[10] Pimm SL, Russell GJ, Gittleman JL, Brooks TM (1995) The future of biodiversity. Science 269: 347–350. 17841251 10.1126/science.269.5222.347

[11] Hubert N, Delrieu-Trottin E, Irisson JO, Meyer C, Planes S (2010) Identifying coral reef fish larvae through DNA barcoding: a test case with the families Acanthuridae and Holocentridae. Mol Phylogenet Evol 55: 1195–1203. 20188843 10.1016/j.ympev.2010.02.023

[12] Bunce M, Worthy TH, Ford T, Hoppitt W, Willerslev E, et al (2003) Extreme reversed sexual size dimorphism in the extinct New Zealand moa *Dinornis* . Nature 425: 172–175. 12968178 10.1038/nature01871

[13] Granjon L, Aniskin VM, Volobouev V, Sicard B (2002) Sand-dwellers in rocky habitats: a new species of *Gerbillus* (Mammalia: Rodentia) from Mali. J Zool 256: 181–190.

[14] Hsieh HM, Huang LH, Tsai LC, Kuo YC, Meng HH, et al (2003) Species identification of rhinoceros horns using the cytochrome *b* gene. Forensic Sci Int 136: 1–11. 10.1016/s0379-0738(03)00251-2 12969614

[15] Hebert P, Cywinska A, Ball SL, deWaard JR (2003) Biological identifications through DNA barcodes. Proc R Soc Lond B 270: 313–321. 10.1098/rspb.2002.2218 PMC1691236 12614582

[16] Teletchea F (2010) After 7 years and 1000 citations: Comparative assessment of the DNA barcoding and the DNA taxonomy proposals for taxonomists and non-taxonomists. Mitochondrial DNA 21: 206–226. 21171865 10.3109/19401736.2010.532212

[17] Harris JD (2003) Can you bank on GenBank? Trends Ecol Evol 18: 317–319.

[18] Télétchéa F, Bernillon J, Duffraisse M, Laudet V, Hänni C (2008) Molecular identification of vertebrate species by oligonucleotide microarray in food and forensic samples. J Appl Ecol 45: 967–975.

[19] Bradley R, Baker R (2001) A test of the genetic species concepts: cytochrome-b sequences and mammals. J Mammal 82: 960–973.

[20] Rubinoff D, Cameron S, Will K (2006) A genomic perspective on the shortcomings of mitochondrial DNA for “barcoding” identification. J Hered 97: 581–594. 17135463 10.1093/jhered/esl036

[21] Bensasson D, Zhang D, Hartl DL, Hewitt GM (2001) Mitochondrial pseudogenes: evolution's misplaced witnesses. Trends Ecol Evol 16: 314–321. 11369110 10.1016/s0169-5347(01)02151-6

[22] Richly E, Leister D (2004) NUMTs in sequenced eukaryotic genomes. Mol Biol Evol 21: 1081–1084. 15014143 10.1093/molbev/msh110

[23] Dubey S, Michaux J, Brunner H, Hutterer R, Vogel P (2009) False phylogenies on wood mice due to cryptic cytochrome-*b* pseudogene. Mol Phylogenet Evol 50: 633–641. 19126432 10.1016/j.ympev.2008.12.008

[24] Waits LP, Paetkau D (2005) Noninvasive genetic sampling tools for wildlife biologists: a review of applications and recommendations for accurate data collection. J Wildl Manage 69: 1419–1433.

[25] Hajibabaei M, Shokralla S, Zhou X, Singer GA, Baird DJ (2011) Environmental barcoding: a next-generation sequencing approach for biomonitoring applications using river benthos. PLoS ONE 6: e17497. 21533287 10.1371/journal.pone.0017497 PMC3076369

[26] Metzker ML (2010) Sequencing technologies - the next generation. Nat Rev Genet 11: 31–46. 19997069 10.1038/nrg2626

[27] Hajibabaei M, Singer GA, Clare EL, Hebert PD (2007) Design and applicability of DNA arrays and DNA barcodes in biodiversity monitoring. BMC Biol 5: 24. 17567898 10.1186/1741-7007-5-24 PMC1906742

[28] Meusnier I, Singer GA, Landry JF, Hickey DA, Hebert PD, et al (2008) A universal DNA mini-barcode for biodiversity analysis. BMC Genomics 9: 214. 18474098 10.1186/1471-2164-9-214 PMC2396642

[29] Taberlet P, Camarra JJ, Griffin S, Uhres E, Hanotte O, et al (1997) Noninvasive genetic tracking of the endangered Pyrenean brown bear population. Mol Ecol 6: 869–876. 9301075

[30] Murphy MA, Waits LP, Kendall KC (2000) Quantitative evaluation of fecal drying methods for brown bear DNA analysis. Wildl Soc Bull 951–957.

[31] Galan M, Guivier E, Caraux G, Charbonnel N, Cosson JF (2010) A 454 multiplex sequencing method for rapid and reliable genotyping of highly polymorphic genes in large-scale studies. BMC Genomics 11: 296. 20459828 10.1186/1471-2164-11-296 PMC2876125

[32] Margulies M, Egholm M, Altman WE, Attiya S, Bader JS, et al (2005) Genome sequencing in microfabricated high-density picolitre reactors. Nature 437: 376–380. 16056220 10.1038/nature03959 PMC1464427

[33] Musser G, Carleton M (2005) Superfamily Muroidea. In Mammal species of the world. A taxonomic and geographic reference Volume 2. 3rd edition. Edited by: Wilson DE, Reeder DM. Baltimore, Johns Hopkins University. 894–1531.

[34] Lecompte E, Brouat C, Duplantier JM, Galan M, Granjon L, et al (2005) Molecular identification of four cryptic species of *Mastomys* (Rodentia, Murinae). Biochem Syst Ecol 33: 681–689.

[35] Ben Faleh A, Cosson J, Tatard C, Ben Othmen A, Said K, et al (2010) Are there two cryptic species of the lesser jerboa *Jaculus jaculus* (Rodentia: Dipodidae) in Tunisia? Evidence from molecular, morphometric, and cytogenetic data. Biol J Linn Soc Lond 99: 673–686.

[36] Jenkins PD, Kilpatrick W, Robinson M, Timmins R (2004) Morphological and molecular investigations of a new family, genus and species of rodent (Mammalia: Rodentia: Hystricognatha) from Lao PDR. System Biodivers 2: 419–454.

[37] Musser G, Smith A, Robinson MF, Lunde D (2005) Description of a new genus and species of rodent (Murinae, Muridae, Rodentia) from the Khammouan limestone national biodiversity conservation area in Lao PDR. American Museum novitates 3497: 1–31.

[38] Helgen KM (2005) A new species of murid rodent (genus *Mayermys*) from South-eastern New Guinea. Mammalian Biology 70: 61–67.

[39] Musser G, Lunde D, Truong Son N (2006) Description of a new genus and species of rodent (Murinae, Muridae, Rodentia) from the lower karst region of Northeastern Vietnam. American Museum novitates 3571: 1–41.

[40] Meerburg BG, Singleton GR, Kijlstra A (2009) Rodent-borne diseases and their risks for public health. Crit Rev Microbiol 35: 221–270. 19548807 10.1080/10408410902989837

[41] Pagès M, Chaval Y, Herbreteau V, Waengsothorn S, Cosson JF, et al (2010) Revisiting the taxonomy of the Rattini tribe: a phylogeny-based delimitation of species boundaries. BMC Evol Biol 10: 184. 20565819 10.1186/1471-2148-10-184 PMC2906473

[42] Meglecz E, Piry S, Desmarais E, Galan M, Gilles A, et al (2011) SESAME (SEquence Sorter & AMplicon Explorer): genotyping based on high-throughput multiplex amplicon sequencing. Bioinformatics 27: 277–278. 21084284 10.1093/bioinformatics/btq641 PMC3018808

[43] Gilles A, Meglecz E, Pech N, Ferreira S, Malausa T, et al (2011) Accuracy and quality assessment of 454 GS-FLX Titanium pyrosequencing. BMC Genomics 12: 245. 21592414 10.1186/1471-2164-12-245 PMC3116506

[44] Lecompte E, Denys C, Granjon L (2005) Confrontation of morphological and molecular data: the *Praomys* group (Rodentia, Murinae) as a case of adaptive convergences and morphological stasis. Mol Phylogenet Evol 37: 899–919. 16111900 10.1016/j.ympev.2005.06.018

[45] Jaarola M, Martinkova N, Gunduz I, Brunhoff C, Zima J, et al (2004) Molecular phylogeny of the speciose vole genus *Microtus* (Arvicolinae, Rodentia) inferred from mitochondrial DNA sequences. Mol Phylogenet Evol 33: 647–663. 15522793 10.1016/j.ympev.2004.07.015

[46] Aplin K, Suzuki H, Chinen AA, Chesser T, ten Have J, et al (2011) Multiple geographic origins of commensalism and complex dispersal history of black rats. PLoS ONE 6: e26357. 22073158 10.1371/journal.pone.0026357 PMC3206810

[47] Ndiaye A, Bâ K, Aniskin VM, Benazzou T, Chevret P, et al (2011) Evolutionary systematics and biogeography of endemic gerbils (Rodentia, Muridae) from Morocco: an integrative approach. Zool Scr 41: 11–28.

[48] Nicolas V, Granjon L, Duplantier JM, Cruaud C, Dobigny G (2009) Phylogeography of spiny mice (genus *Acomys*, Rodentia: Muridae) from the south-western margin of the Sahara with taxonomic implications. Biol J Linn Soc Lond 98: 26–46.

[49] Haynes S, Jaarola M, Searle JB (2003) Phylogeography of the common vole (*Microtus arvalis*) with particular emphasis on the colonization of the Orkney archipelago. Mol Ecol 12: 951–956. 12753214 10.1046/j.1365-294x.2003.01795.x

[50] DeWoody JA, Chesser RK, Baker RJ (1999) A translocated mitochondrial cytochrome *b* pseudogene in voles (Rodentia: *Microtus*). J Mol Evol 48: 380–382. 10093228 10.1007/pl00013154

[51] Triant DA, DeWoody JA (2008) Molecular analyses of mitochondrial pseudogenes within the nuclear genome of arvicoline rodents. Genetica 132: 21–33. 17333478 10.1007/s10709-007-9145-6

[52] Triant DA, DeWoody JA (2009) Demography and phylogenetic utility of Numt pseudogenes in the Southern red-backed vole (*Myodes gapperi*). J Mammal 90: 561–570.

[53] Pagès M, Chevret P, Gros-Balthazard M, Hughes S, Alcover JA, et al (accepted) Ancient DNA analysis reveals unsuspected phylogenetic affinities between mice and the extinct *Malpaisomys insularis* (Rodentia, Murinae), an endemic to the Canary Islands. PLoS ONE 7: e31123. 22363563 10.1371/journal.pone.0031123 PMC3283599

[54] Miraldo A, Hewitt GM, Dear PH, Paulo OS, Emerson BC (2012) Numts help to reconstruct the demographic history of the ocellated lizard (*Lacerta lepida*) in a secondary contact zone. Mol Ecol 21: 1005–1018. 22221514 10.1111/j.1365-294X.2011.05422.x

[55] Quéré JP, Le Louarn H (2011) Les rongeurs de France. Faunistique et biologie. 3e édition revue et augmentée. Editions Quae, Collection Guide pratique, 312 p.

[56] Michaux JR, Chevret P, Filippucci MG, Macholan M (2002) Phylogeny of the genus *Apodemus* with a special emphasis on the subgenus *Sylvaemus* using the nuclear IRBP gene and two mitochondrial markers: cytochrome *b* and 12S rRNA. Mol Phylogenet Evol 23: 123–136. 12069545 10.1016/S1055-7903(02)00007-6

[57] Galewski T, Tilak MK, Sanchez S, Chevret P, Paradis E, et al (2006) The evolutionary radiation of Arvicolinae rodents (voles and lemmings): relative contribution of nuclear and mitochondrial DNA phylogenies. BMC Evol Biol 6: 80. 17029633 10.1186/1471-2148-6-80 PMC1618403

[58] Robins J, Hingston M, Matisoo-Smith E, Ross H (2007) Identifying *Rattus* species using mitochondrial DNA. Mol Ecol Notes 7: 717–729.

[59] Robins JH, McLenachan PA, Phillips MJ, Craig L, Ross HA, et al (2008) Dating of divergences within the *Rattus* genus phylogeny using whole mitochondrial genomes. Mol Phylogenet Evol 49: 460–466. 18725306 10.1016/j.ympev.2008.08.001

[60] Rowe KC, Aplin KP, Baverstock PR, Moritz C (2011) Recent and rapid speciation with limited morphological disparity in the genus *Rattus* . Syst Biol 60: 188–203. 21239388 10.1093/sysbio/syq092

[61] Rowe KC, Reno ML, Richmond DM, Adkins RM, Steppan SJ (2008) Pliocene colonization and adaptive radiations in Australia and New Guinea (Sahul): Multilocus systematics of the old endemic rodents (Muroidea: Murinae). Mol Phylogenet Evol 47: 84–101. 18313945 10.1016/j.ympev.2008.01.001

[62] Latinne A, Waengsothorn S, Herbreteau V, Michaux J (2011) Evidence of complex phylogeographic structure for the threatened rodent *Leopoldamys neilli*, in Southeast Asia. Conserv Genet 12: 1495–1511.

[63] Dobigny G, Lecompte E, Tatard C, Gauthier P, Bâ K, et al (2008) An update on the taxonomy and geographic distribution of the cryptic species *Mastomys kollmannspergeri* (Muridae, Murinae) using combined cytogenetic and molecular data. J Zool 276: 368–374.

[64] Lecompte E, Aplin K, Denys C, Catzeflis F, Chades M, et al (2008) Phylogeny and biogeography of African Murinae based on mitochondrial and nuclear gene sequences, with a new tribal classification of the subfamily. BMC Evol Biol 8: 199. 18616808 10.1186/1471-2148-8-199 PMC2490707

[65] Colangelo P, Granjon L, Taylor PJ, Corti M (2007) Evolutionary systematics in African gerbilline rodents of the genus *Gerbilliscus*: inference from mitochondrial genes. Mol Phylogenet Evol 42: 797–806. 17113792 10.1016/j.ympev.2006.10.001

[66] Ducroz JF, Volobouev V, Granjon L (1998) A molecular perspective on the systematics and evolution of the genus *Arvicanthis* (Rodentia, Muridae): inferences from complete cytochrome *b* gene sequences. Mol Phylogenet Evol 10: 104–117. 9751921 10.1006/mpev.1997.0477

[67] Chevret P, Dobigny G (2005) Systematics and evolution of the subfamily Gerbillinae (Mammalia, Rodentia, Muridae). Mol Phylogenet Evol 35: 674–688. 15878135 10.1016/j.ympev.2005.01.001

[68] Taberlet P, Waits LP, Luikart G (1999) Noninvasive genetic sampling: look before you leap. Trends Ecol Evol 14: 323–327. 10407432 10.1016/s0169-5347(99)01637-7

[69] Köhn W, Wayne RK (1997) Facts from feces revisited. Trends Ecol Evol 12: 223–227. 21238046 10.1016/s0169-5347(97)01050-1

[70] Paabo S, Poinar H, Serre D, Jaenicke-Despres V, Hebler J, et al (2004) Genetic analyses from ancient DNA. Annu Rev Genet 38: 645–679. 15568989 10.1146/annurev.genet.37.110801.143214

[71] Hofreiter M, Jaenicke V, Serre D, Haeseler Av A, Paabo S (2001) DNA sequences from multiple amplifications reveal artifacts induced by cytosine deamination in ancient DNA. Nucleic Acids Res 29: 4793–4799. 11726688 10.1093/nar/29.23.4793 PMC96698

[72] Gilbert MT, Hansen AJ, Willerslev E, Rudbeck L, Barnes I, et al (2003) Characterization of genetic miscoding lesions caused by postmortem damage. Am J Hum Genet 72: 48–61. 12489042 10.1086/345379 PMC420012

[73] Granjon L, Duplantier JM (2009) Les rongeurs de l'Afrique sahélo-soudanienne. IRD Editions et Publications scientifiques du Muséum, Collection Faune et Flore Tropicales 43: 215.

[74] Shehzad W, Riaz T, Nawaz MA, Miquel C, Poillot C, et al (2012) Carnivore diet analysis based on next-generation sequencing: application to the leopard cat (*Prionailurus bengalensis*) in Pakistan. Mol Ecol 21: 1951–1965. 22250784 10.1111/j.1365-294X.2011.05424.x

[75] Granjon L, Bruderer C, Cosson JF, Dia AT, Colas F (2002) The small mammal community of a coastal site of south-west Mauritania. Afri J Ecol 40: 10–17.

[76] Thiam M, Bâ K, Duplantier JM (2008) Impacts of climatic changes on small mammal communities in the Sahel (West Africa) as evidenced by owl pellet analysis. Afr Zool 43: 135–143.

[77] Bohmann K, Monadjem A, Lehmkuhl Noer C, Rasmussen M, Zeale MR, et al (2011) Molecular diet analysis of two african free-tailed bats (Molossidae) using high throughput sequencing. PLoS ONE 6: e21441. 21731749 10.1371/journal.pone.0021441 PMC3120876

[78] Hall TA (1999) BioEdit: a user-friendly biological sequence alignment editor and analysis program for Windows 95/98/NT. Nucleic Acids Symposium Series 41: 95–98.

[79] Tamura K, Petersen D, Petersen N, Stecher G, Nei M, et al (2011) MEGA5: Molecular Evolutionary Genetics Analysis using Maximum Likelihood, Evolutionary Distance, and Maximum Parsimony Methods. Mol Biol Evol 28: 2731–2739. 21546353 10.1093/molbev/msr121 PMC3203626

[80] Winnepenninckx B, Backeljau T, De Wachter R (1993) Extraction of high molecular weight DNA from molluscs. Trends Genet 9: 407. 8122306 10.1016/0168-9525(93)90102-n

[81] Gilbert MT, Bandelt HJ, Hofreiter M, Barnes I (2005) Assessing ancient DNA studies. Trends Ecol Evol 20: 541–544. 16701432 10.1016/j.tree.2005.07.005

[82] Huse SM, Huber JA, Morrison HG, Sogin ML, Welch DM (2007) Accuracy and quality of massively parallel DNA pyrosequencing. Genome Biol 8: R143. 17659080 10.1186/gb-2007-8-7-r143 PMC2323236

[83] Zhang Z, Schwartz S, Wagner L, Miller W (2000) A greedy algorithm for aligning DNA sequences. J Comput Biol 7: 203–214. 10890397 10.1089/10665270050081478

[84] Piry S, Guivier E, Realini A, Martin JF (2012) |SE|S|AM|E| Barcode: a NGS-oriented software for amplicon characterization – application to species and environmental barcoding. Mol Ecol Resour 12 doi:10.1111/j.1755-0998.2012.03171.x 10.1111/j.1755-0998.2012.03171.x 22823139

[85] Stamatakis A (2006) RAxML-VI-HPC: maximum likelihood-based phylogenetic analyses with thousands of taxa and mixed models. Bioinformatics 22: 2688–2690. 16928733 10.1093/bioinformatics/btl446

[86] Lanave C, Preparata G, Saccone C, Serio G (1984) A new method for calculating evolutionary substitution rates. J Mol Evol 20: 86–93. 6429346 10.1007/BF02101990

[87] Yang Z (1994) Maximum likelihood phylogenetic estimation from DNA sequences with variable rates over sites: approximate methods. J Mol Evol 39: 306–14. 7932792 10.1007/BF00160154

[88] Stamatakis A, Hoover P, Rougemont J (2008) A rapid bootstrap algorithm for the RAxML Web servers. Syst Biol 57: 758–771. 18853362 10.1080/10635150802429642

[89] Kimura M (1980) A simple method for estimating evolutionary rates of base substitutions through comparative studies of nucleotide sequences. J Mol Evol 16: 111–20. 7463489 10.1007/BF01731581

